# Lipid nanoparticles silence tumor necrosis factor α to improve wound healing in diabetic mice

**DOI:** 10.1002/btm2.10123

**Published:** 2018-12-20

**Authors:** Lisa N. Kasiewicz, Kathryn A. Whitehead

**Affiliations:** ^1^ Dept. of Chemical Engineering Carnegie Mellon University 5000 Forbes Avenue, Pittsburgh PA 15213; ^2^ Dept. of Biomedical Engineering Carnegie Mellon University 5000 Forbes Avenue, Pittsburgh PA 15213

**Keywords:** chronic inflammation, diabetic ulcer, lipid nanoparticles, lipidoid, siRNA, TNFα, wound healing

## Abstract

Diabetes mellitus is a mounting concern in the United States, as are the mortality and morbidity that result from its complications. Of particular concern, diabetes patients frequently suffer from impaired wound healing and resultant nonhealing diabetic foot ulcers. These ulcers overproduce tumor necrosis factor α (TNFα), which reduces wound bed cell migration and proliferation while encouraging apoptosis. Herein, we describe the use of siRNA‐loaded lipid nanoparticles (LNPs) as a potential wound treatment to combat an overzealous immune response and facilitate wound closure. LNPs were formulated with an ionizable, degradable lipidoid and siRNA specific for TNFα. Topical application of nanoparticles reduced TNFα mRNA expression in the wound by 40–55% in diabetic and nondiabetic mice. In diabetic mice, this TNFα knockdown accelerated wound healing compared to untreated controls. Together, these results serve as proof‐of‐concept that RNA interference therapy using LNPs can reduce the severity and duration of chronic diabetic wounds.

## 1 INTRODUCTION

Diabetes is becoming a major crisis for the health‐care industry. As the population ages, the number of those affected is rising, with 50% of Americans now suffering from prediabetes or diabetes.^1^ Approximately, 90% of diabetic people suffer from Type II diabetes, with many individuals unaware that they have it. This delays early treatment and intervention, worsening the chance of developing a constellation of negative complications.^2^ These complications include ischemia, peripheral neuropathy, atherosclerosis, kidney failure, and impaired wound healing. This study addresses the latter, as delayed wound healing can result in the formation of a chronic foot ulcer in up to 25% of diabetics. Unfortunately, chronic foot ulcers can lead to lower limb amputation,^3^ which does not address wound pathology and has a 3‐year survival rate of only 50%. As such, there is an imperative need for a treatment that addresses the inflammatory pathology of the diabetic wound bed in a way that prevents the formation of the ulcer.^4^

Unlike acute wounds, diabetic foot ulcers do not proceed normally through the three general stages of wound healing: inflammation, re‐epithelization, and wound remodeling.^5^ Instead, diabetic wound bed cells undergo harmful phenotypic changes that impair their ability to react appropriately to the normal cytokine and growth factor cascade.^6^, ^7^ In addition, higher than normal numbers of inflammatory macrophages have been shown to reside within diabetic wounds, where they overproduce tumor necrosis factor alpha (TNFα), one of the most important inflammatory cytokines.^8^, ^9^, ^10^, ^11^ In normal wound healing, a small, tightly controlled amount of TNFα is required for fibroblast migration, proliferation, and remodeling.^12^ When overproduced, however, TNFα damages the wound further by upregulating cellular apoptosis, reactive oxygen species production, and matrix degradation.^9^, ^10^, ^13^, ^14^, ^15^ Currently, the most common method of treatment for diabetic wounds is the use of moisture‐retentive bandages, hyperbaric oxygen therapy, and antibiotics.^16^, ^17^ These treatments are often ineffective, as they do not address the chronic inflammation and other biological irregularities that caused the ulcer to form. Biomaterials research has focused on correcting these irregularities with an array of nanoparticles, hydrogels, nanofibrous meshes, and dressings that deliver drugs (e.g., growth factors) to the wound.^18^

Alternatively, it may be possible to treat diabetic wounds using short interfering RNA, which can reduce the expression of problematic proteins.^19^, ^20^ Although RNA interference is a promising therapeutic strategy, its use in wound healing has been limited. There have been several reports of improved wound healing outcomes upon gene silencing of matrix metalloproteinases (MMPs)^21^, ^22^, ^23^ and prolyl hydroxylase domain protein 2.^24^, ^25^ In separate studies, siRNA loaded in hydrogel dressings has induced modest knockdown of other gene targets, including xanthine dehydrogenase^26^ and the tumor suppressor gene, p53,^27^ in the diabetic wound.

In this study, we use RNA interference to examine how reduction in the inflammatory cytokine, TNFα, influences the wound healing process. Sufferers of other inflammatory diseases, like inflammatory bowel disease and rheumatoid arthritis, have benefited from treatment with antagonists of TNFα.^28^, ^29^, ^30^ Diabetic wounds have improved with systemic anti‐TNFα therapies^31^ and antibody‐based neutralization^32^ but with an increased risk of infection and severe liver damage.^30^, ^32^, ^33^, ^34^, ^35^ Local downregulation of TNFα has the potential to correct chronic inflammation in the wound while avoiding the negative side effects associated with systemic suppression of TNFα.

Delivery of siRNA, whether systemically or topically, requires a vehicle that protects the RNA cargo and enables its transport across cell and endosomal membranes into the cytoplasm of target cells.^19^ Fortunately, we have previously developed LNPs that potently deliver siRNA in vivo^36^, ^37^, ^38^ and control inflammatory feedback loops via TNFα silencing in a macrophage‐fibroblast co‐culture model.^39^ Herein, we show that LNPs can be topically delivered in solution to nondiabetic and diabetic mouse wounds to silence TNFα and improve wound healing outcomes.

## 2 MATERIALS AND METHODS

### 2.1 Nanoparticle formulation

Nanoparticles were formulated with the three‐tailed version of lipidoid 306O_13_,^36^ which was synthesized via the Michael addition of 3,3′‐diaminodipropylamine (Acros Organics) to tridecyl acrylate (Pfaltz and Bauer) at a stoichiometric ratio of 1:3 as described previously.^37^, ^39^ The lipidoid was then purified over a silica column on a Teledyne ISCO chromatography system to isolate the three‐tailed product. For nanoparticle formulation, a lipid solution was formed by mixing the lipidoid, distearoyl‐sn‐glycerol‐3‐phosphocholine (DSPC, Avanti Polar Lipids, Alabaster, Alabama), cholesterol (Sigma Aldrich), and C14‐PEG (Avanti Polar Lipids) at a molar ratio of 50:10:38.5:1.5 in ethanol (Sigma Aldrich) and 10 mM citrate buffer. Silencer Select Pre‐Designed siRNA against TNFα (s75248, Thermo Fisher) was diluted in 10 mM sodium citrate and combined with the lipid solution at a final lipidoid to siRNA weight ratio of 5:1. The solution was vortexed after each reagent addition, and the lipid solution was added to the siRNA solution before being diluted to the desired final concentration in phosphate buffered saline (PBS).

### 2.2 Nanoparticle characterization

Nanoparticles were diluted to a final siRNA concentration of 1 μg/mL in PBS. Percent siRNA entrapment was determined via the Quant‐iT Ribogreen assay (Invitrogen) according to manufacturer instructions. Nanoparticle size was measured with a Malvern Zetasizer Nano (Malvern Instruments, UK).

### 2.3 Animal studies

All mouse experiments were approved by the Institutional Animal Care and Use Committee (IACUC) at Carnegie Mellon University. C57BL/6 mice were either purchased from Charles River Laboratories (Wilmington, MA) or obtained from an institutionally managed breeding colony. Genetically diabetic *BKS.Cg*‐*Dock7* ^*m*^ +/+ *Lepr* ^*db*^/*J* mice were purchased from the Jackson Laboratory (Bar Harbor, ME). Mice were housed in cages of fewer than six animals, with controlled temperature (25°C), 12‐hr light–dark cycles, and free access to food and water. Mice used in this study were males between the ages of 10 and 16 weeks. Prior to wounding, the backs of the mice were shaved with electric clippers and circle templates 8 mm in diameter were traced on both the right and left flanks. Surgery was performed under anesthesia and 3 mg/kg bupivacaine was injected in a line block formation around each wound site (6 mg/kg total dose). Two 8 mm full thickness excisional wounds were created with surgical scissors using the template circles.

To calculate appropriate topical dosing, we referred to experiments conducted in 24 well plates in a previous study.^39^ Cells in these experiments were dosed at 25 pmol siRNA/cm^2^ well area (100 pmol/cm^3^). In vivo wounds were modeled as a cylinder (radius of 5 mm and height of 1 mm) and treated with an equivalent or greater dose of siRNA than what was used in vitro. Doses were calculated for a volume of 10 μL of nanoparticle solution added topically to the wound and are listed in each experiment. The 10 μL of nanoparticle solution was administered via pipette tip onto the wound and was not injected into the tissue. On average, most of the wound was covered. Administration was performed while the mouse was anesthetized with isoflurane, and the liquid was allowed to sit on the wound for up to 10 min without the mouse moving. By the time the mouse was removed from the isoflurane nose cone and allowed to regain consciousness (a process that took an additional 5 min), the liquid was almost entirely absorbed.

### 2.4 Confocal microscopy

Mice were wounded and immediately received 10 μL of either PBS or 5 μM nanoparticle solution. The nanoparticles used in these studies were loaded with Cy5.5‐labeled siRNA. Mice were sacrificed, and wounds were excised 2 hr after treatment with surgical scissors and immediately fixed in 4% formaldehyde solution. After a period of overnight fixation at 4°C, they were washed with PBS, permeabilized with 0.1% Triton‐X100, and incubated for 2 hr with staining solutions. The staining solution contained DAPI (12 μg/mL, 358 nm/461 nm) to mark nucleic acids, AlexaFluor 488® conjugated phalloidin (5 units/mL, 495 nm/518 nm) to bind actin, and PE‐Texas Red® conjugated F4/80 antibody (0.15 mg/mL, 565 nm/615 nm) to identify macrophages. After staining, the wounds were washed three times with PBS, mounted on glass slides, and placed under coverslips. Prepared slides were imaged at ×63 magnification using a Zeiss LSM 700 confocal microscope with ZEN 2012 SP1 software. Images were captured using a Plan‐Apochromat ×63/1.40 Oil DIC objective and an X‐Cite Series 120Q laser source exposing at 405, 488, and 555 nm. Images were captured at room temperature and represent a single time point. Images were approximately 101.61 μm × 101.61 μm. No additional processing or averaging was performed to enhance the resolution of the images. Image J (NIH) image processing software was used to prepare confocal images.

### 2.5 RNA interference wound healing experiments

Double‐wounded mice were anesthetized 24 hr after wounding for topical application of 10 μL per wound of solution. The right flank wound received a nanoparticle solution (100, 250, and 500 nM doses of siRNA against TNFα), while the left flank wound received PBS. Non‐diabetic double wounded mice were sacrificed 24 hr after treatment. Diabetic mice received a second, identical treatment 48 hr post‐wounding and were then sacrificed either 4 days or 2 weeks after wounding, as indicated. For experiments in which gene silencing was being quantified, excised wounds were immediately placed into 2.0 mL homogenizer tubes filled with 800 μL of TRIzol (Thermo Fisher). The homogenizer tubes were pre‐filled with 0.5 mm garnet shards and a 6.0 mm in diameter zirconium bead (Laboratory Supply Network). A BeadBug tissue homogenizer (Laboratory Supply Network) was used for 120 s at 3000 rpm to homogenize the wounds in TRIzol. The tubes were then stored at −80 °C until RNA extraction.

### 2.6 Wound area analysis

Mice were photographed in the same position next to the same object of known length each day of the experiment. Images were then processed using ImageJ to calculate wound area and the change in wound area for each day.

### 2.7 RNA extraction and cDNA creation

RNA extraction and purification from the homogenized wound tissue was accomplished with TRIzol according to the manufacturer's instructions. RT‐PCR was performed with the High Capacity cDNA Reverse Transcription kit (Applied Biosystems) to form 2 μg of cDNA. The quality and quantity of mRNA and cDNA were assessed by the absorbance at 260/280 nm with the Nanodrop 2000 UV‐Vis spectrophotometer (Thermo Scientific).

### 2.8 Quantitative PCR

qPCR was performed in a 384‐well block on a ViiA™ 7 Real‐Time PCR system machine with purchased Taqman Gene Expression Master Mix (Applied Biosystems), along with GAPDH (Mm99999915_g1) and TNFα (Mm00443258_m1) Taqman probes (Thermo Fisher). About 10 μL of Master Mix, 1 μL of endogenous control probe, and 1 μL of target gene probe were used per well. All runs utilized the comparative Ct method and the following run protocol: 50 °C (2 min), 95 °C (10 min), 40 cycles of 95 °C (15 s) and 60 °C (1 min). qPCR experiments consisted of three to five biological replicates and two technical replicates per each biological. The target gene (TNFα) was normalized to the endogenous control gene (GAPDH) for each sample. For double wounded mice, treated samples were normalized again to the nanoparticle‐free control sample for each mouse.

### 2.9 Statistical analysis

All mean values are expressed as ± standard deviation. Unpaired Student's t‐tests and one‐way anova tests were used where appropriate to evaluate statistical significance. A *p* <.05 was considered significant.

## 3 RESULTS

### 3.1 Lipidoid nanoparticles reduce TNFα gene expression in double‐wounded nondiabetic mice

Previously, we showed that LNPs effectively delivered siRNA and induced TNFα gene silencing in an in vitro co‐culture wound model.^39^ These experiments motivated the present study, in which we assessed the effect of TNFα gene silencing on the wound healing process in mice. Although healthy animals do not require assistance in wound healing, we conducted our first gene silencing studies in nondiabetic mice as a proof‐of‐concept. Standard mice are far less expensive than diabetic mice, and their wounds are populated by the cell types that express TNFα, including macrophages and neutrophils. These experiments were conducted using a double‐wounded mouse model in which mice were wounded on each flank. Although one wound served as a negative control and received phosphate buffered saline (PBS) treatment, the other wound was treated with siRNA‐loaded nanoparticles. By providing a built‐in control wound for each mouse, the double‐wounded model alleviates the issue of inter‐mouse variability in TNFα expression in response to wounding.^40^

**Figure 1:**
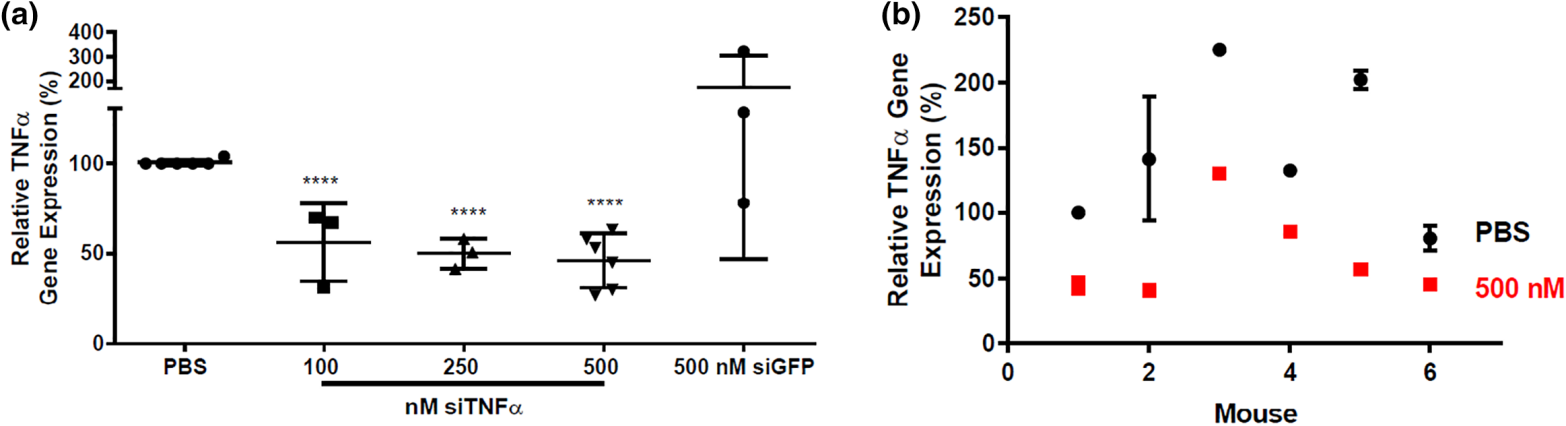
LNPs reduced TNFα gene expression in double‐wounded, nondiabetic mice. Mice received two wounds each. One wound was treated with PBS (control), and the other was treated with LNPs loaded with siRNA against TNFα. (a) LNP treatments with doses between 100 and 500 nM silenced TNFα gene expression between 44 and 54%. Control LNPs containing a 500 nM dose of control siRNA (siGFP) did not induce gene knockdown. All TNFα expression levels are normalized to the untreated wound on each mouse. (b) Although there was significant variability in TNFα expression from mouse to mouse, all mouse wounds treated with 500 nM siTNFα (red squares) experienced reduced TNFα expression compared to PBS‐treated wounds (black circles). Here, each TNFα gene expression is normalized to the PBS‐treated gene expression of mouse #1. Error bars represent s. d. (*n* = 3 − 6)

In these experiments, wounding occurred on Day 1, and mice received a single treatment of siRNA‐loaded nanoparticles and PBS treatment on Day 2. On Day 3, mice were sacrificed and the wound tissue was excised for processing and gene expression analysis. LNP formulations had siRNA entrapment values between 73 and 85% and an average Z‐average diameter of 110 nm. Figure 1a shows the effect of several treatments on TNFα expression, including LNPs loaded with either the target siRNA specific against TNFα or control siRNA specific against green fluorescent protein (GFP). Each wound was dosed with 10 μL of solution. LNPs containing siTNFα and dosed at RNA concentrations between 100 and 500 nM reduced TNFα gene expression between 44 and 54%, with a modest dose‐responsive effect. The LNP control treatment including 500 nM siGFP did not induce gene silencing. Given that one mouse experienced a substantial increase in TNFα expression following control LNP treatment, it is possible that LNPs may cause a mild inflammatory response in some wounds. Together, these data confirm that LNPs are capable of mediating TNFα gene silencing in the wounds of healthy mice.

Figure 1b is an alternative representation of the compressed 500 nM siTNFα data set from Figure 1a. These data, which are normalized to the PBS control wound of mouse 1, are shown to illustrate the variation in TNFα gene expression across six mice (Figure 1b). TNFα expression in PBS‐treated wounds, which, here, varied up to threefold, showed that some mice produced more TNFα in wounds than others in response to wounding. In general, TNFα expression after siRNA‐mediated gene silencing tracked with PBS samples and also varied up to threefold, with silencing ranging from 37% to 74%. These data illustrate the importance of a double‐wounded model in accounting for the intrinsic variability of cytokine response in individual mice.

**Figure 2:**
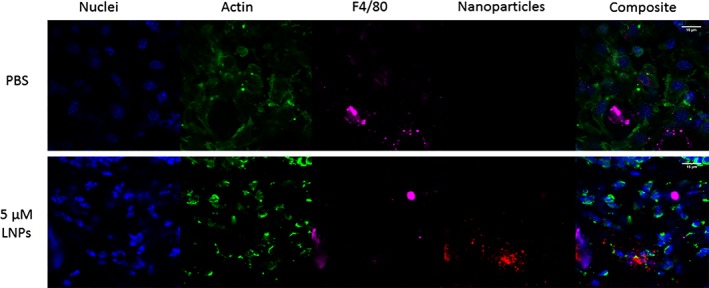
Nanoparticles localize in and around wound bed cells. Wounds on C57BL/6 mice were treated with either PBS (control) or LNPs loaded with Cy5.5‐labeled siRNA at a concentration of 5 μM. Wounds were excised 2 hr post‐treatment and stained for DNA (blue), actin (green), and the F4/80 receptor on macrophages (magenta). Nanoparticles containing siRNA (red) localized within the wound bed without clear affinity for any particular cell type. The scale bar is 15 μm

In addition to confirming that LNPs facilitate gene silencing in mouse wounds, we were interested in observing the localization of LNPs within the wound bed using confocal microscopy. These experiments were challenging because wounds are riddled with dead and dying cells and other debris, and it is difficult to keep them intact upon excision. Wounds were treated with 10 μL of either PBS (negative control) or a 5 μM solution of LNPs encapsulating Cy5.5‐labeled siRNA (Figure 2). Wounds were excised 2 hr post‐treatment and stained with DAPI, phalloidin, and an anti‐F4/80 antibody, which enabled visualization of nuclei, actin filaments, and macrophages, respectively. Macrophages were interspersed throughout the wound bed. LNPs visibly embedded within the wound tissue, with no clear specificity for any cell type. It is possible that nanoparticles localized in a pattern that corresponded to wound bed topology.

### 3.2 TNFα silencing accelerates wound healing in diabetic mice

After showing that TNFα knockdown was possible, we sought to treat diabetic mouse wounds with siRNA‐loaded LNPs. These experiments used *BKS.Cg*‐*Dock7* ^*m*^ +/+ *Lepr* ^*db*^/*J* mice, which are homozygous for the diabetes spontaneous gene mutation, *Lepr* ^*db*^. These mice become Type II diabetic by 4–8 weeks of age and exhibit the hallmarks of Type II Diabetes in humans, including hyperglycemia, obesity, peripheral neuropathy, myocardial disease, and impaired wound healing. As such, they are a common model in the study of diabetic ulcer treatments.^23^, ^41^, ^42^, ^43^, ^44^, ^45^

Two sets of experiments were carried out to probe the efficacy of topically applied, siTNFα‐loaded LNPs. The first set quantified TNFα gene silencing in the initial inflammation phase, requiring mouse sacrifice on Day 4. The second set allowed the wounds to close to completion to study the effect of nanoparticle treatment on wound contraction and healing time. In the first set of experiments, five 12‐week‐old mice were wounded with two flank wounds (Day 1). Twenty‐four hours later on Day 2, they were topically dosed with 10 μL of PBS on their left flank and 10 μL of 250 nM siTNFα encapsulated in LNPs on their right flank. Nanoparticles had a Z‐average diameter of 108 nm and an siRNA entrapment of 75%. Wounds received a second, equivalent treatment on Day 3 and were harvested for RNA processing on Day 4.

**Figure 3:**
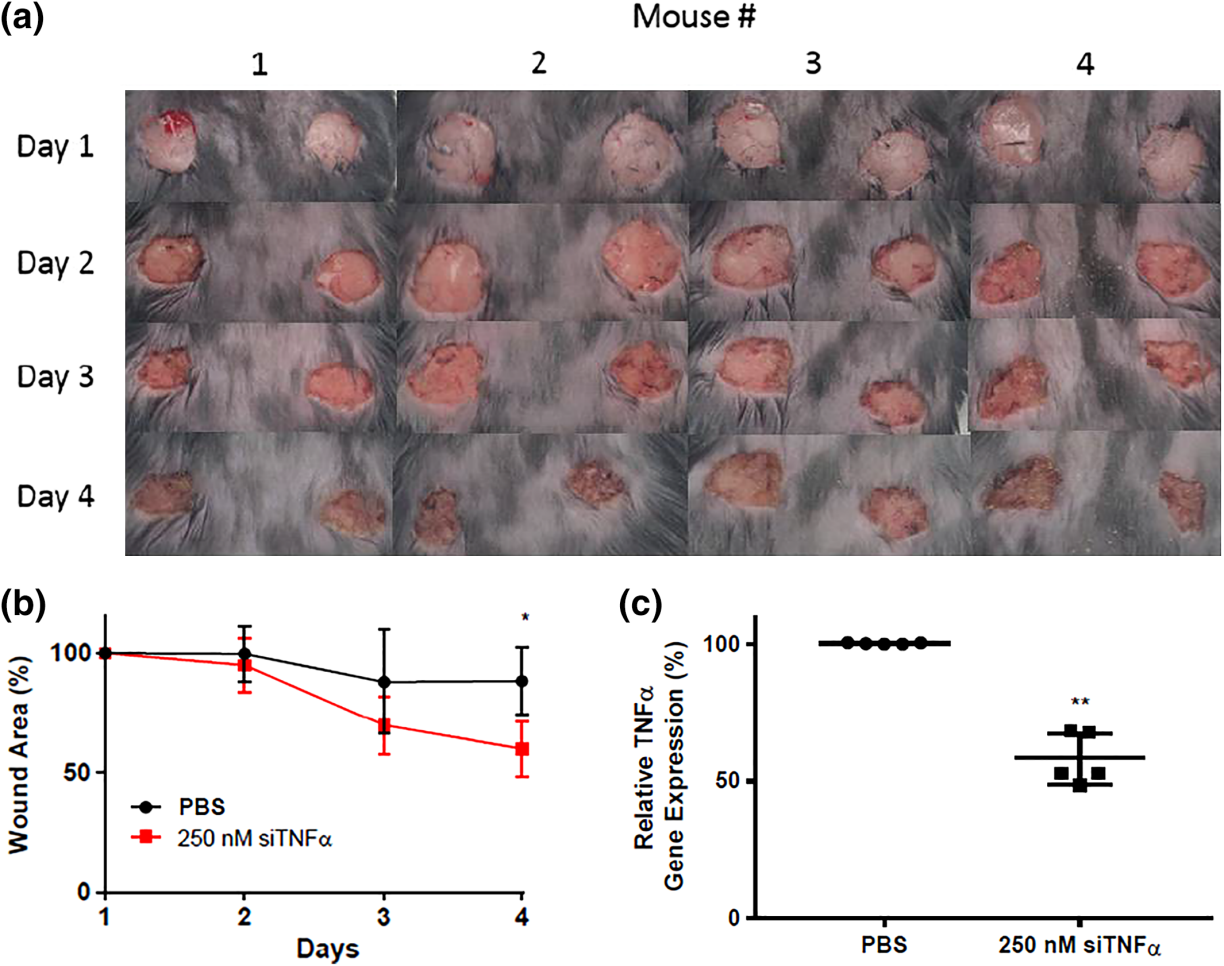
LNP‐mediated TNFα gene silencing accelerated wound healing in diabetic mice. (a) Mice were double‐wounded on Day 1 and treated on Days 2 and 3 with either PBS (control, left wounds) or LNPs containing siTNFα (250 nM, right wounds). Mice were sacrificed on Day 4. (b) The wound area is represented as a percent of its original size. By Day 4, nanoparticle‐treated wounds are statistically significantly smaller than PBS‐treated wounds. (c) Wounds treated with lipid nanoparticles containing siRNA specific against TNFα (siTNFα) experienced a 43% reduction in TNFα expression compared to PBS‐treated control wounds. In each panel, error bars represent s. d (*n* = 4–5, *p < .05, **p < .01)

Wounds were photographed on each day (Figure 3a), and their areas were quantified with ImageJ. The change in wound size over time was calculated by subtracting the wound area for each treatment group from the wound's initial area and normalizing the difference to the initial area (shown in Figure 3b). Here, 100% represents the area of the wound on Day 1. Although both sets of wounds began closing within 4 days, the PBS‐treated wounds contracted at a slower rate than nanoparticle‐treated wounds. By Day 4, the difference in percentage wound closure between the two groups was statistically significant. No side effects such as redness or irritation were observed on any of the treated wounds compared to untreated wounds.

This increased rate of wound contraction for nanoparticle‐treated wounds is reflected in the silencing data. After excision on Day 4, the wounds were immediately processed, and their TNFα gene expression was analyzed by qPCR. Wounds treated with siTNFα‐loaded LNPs experienced a 43% reduction in TNFα compared to control wounds (Figure 3c).

**Figure 4:**
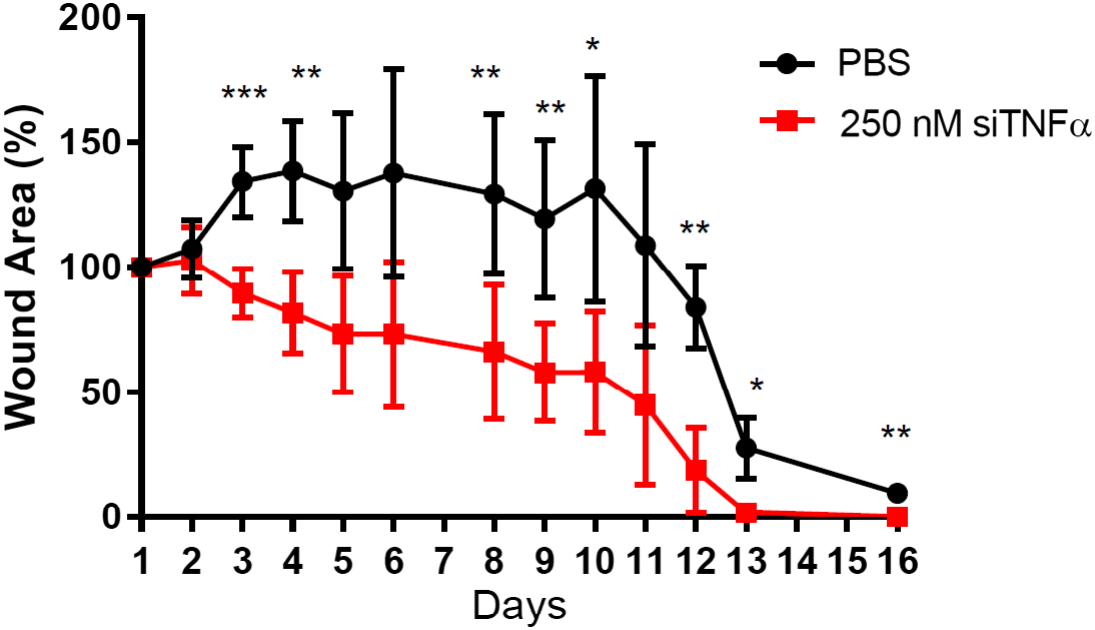
Treatment of diabetic mouse wounds with siRNA‐loaded lipid nanoparticles enabled complete wound healing within 13 days. Mice were wounded on Day 1 and dosed with either PBS (black circles, control) or LNPs containing siRNA specific against TNFα (red squares) on Days 2 and 3. LNP treatment accelerated wound healing over a period of 2 weeks and closed wounds by Day 13 in three of the five mice. The remaining two mice required sacrifice prior to complete treated wound closure. Error bars represent s.d. significance is compared to PBS treated wounds on each day (*n* = 5, *p < .05, **p < .01, ***p < .001)

A second set of experiments assessed the effect of nanoparticle treatment on wound healing to completion. Double‐wounded diabetic mice were treated as described above (once on Day 2 and again on Day 3), and their wounds were photographed every day until the treated wound closed or the mice met the criteria for sacrifice (due to the control wound). Differences between wounds treated with PBS versus 250 nM siTNFα‐loaded LNPs were observed as early as Day 3 (Figure 4). Although nanoparticle‐treated wounds began to close, PBS‐treated wounds worsened as a result of diabetes pathology. By Day 11, nanoparticle‐treated wounds had reduced in area by more than 50%. Unfortunately, two mice were sacrificed on Day 11 because their control wounds had worsened significantly. By Day 13, in the three remaining mice, the nanoparticle‐treated wounds had completely healed. PBS‐treated wounds remained open as of Day 16. Together, these data show that two doses of siTNFα ‐loaded LNPs to diabetic wounds induces ~45% knockdown of TNFα, accelerates healing, and mitigates mortality.

## 4 DISCUSSION

Although siRNA is promising as a therapeutic, its use is often complicated by its large molecular size, negative charge, and the need for delivery vehicles to escape the endosome in sufficient numbers.^19^ For these reasons, siRNA has been utilized only sporadically in literature studies of diabetic wound treatment and is usually delivered with polymer nanoparticles, nanofibrous meshes, or hydrogels.^21^, ^22^, ^23^, ^24^, ^25^, ^26^, ^27^ The most common gene targets include members of the matrix metalloproteinase family, as these enzymes prevent healthy tissue reconstruction when upregulated in diabetic ulcers. Although this is an important avenue of treatment, addressing the chronic inflammation endemic to diabetic foot ulcers represents another promising method.

Other inflammatory disease models, including plaque psoriasis and arthritis, have utilized polymer carriers for siTNFα to reduce chronic inflammation to good effect.^46^, ^47^ Some diabetic mouse studies have also used systemic anti‐TNFα treatments, like neutralizing antibodies,^9^, ^32^, ^48^ to hasten wound healing. To date, however, none have examined the effect of a topically applied compound intended to downregulate TNFα. In this regard, the specificity and local activity of siRNA makes it an ideal choice.

LNPs are among the most efficacious of existing RNA delivery systems.^49^, ^50^, ^51^ Nanoparticles formulated with lipidoid, a type of ionizable lipid‐like material, have been shown to potently deliver siRNA in vivo to several cell types, including hepatocytes, epithelial cells, and difficult to transfect cell lines, like immune cells.^38^, ^39^, ^52^, ^53^, ^54^ To transfect wound bed cells, lipidoid nanoparticles must remain in the wound tissue without being degraded by enzymes or becoming stuck in wound debris. Confocal images show that they remained in the tissue, most likely conforming to wound bed topology, and avoided degradation long enough to be taken up by cells (Figure 2).

Topical application of lipidoid nanoparticles resulted in approximately 50% TNFα gene silencing within the diabetic wound (Figure 3c). This knockdown brought TNFα expression almost down to baseline levels typical of a normoglycemic mouse. It has been shown that TNFα mRNA levels in wounded diabetic mice can be up to three times as high as wounded normoglycemic animals.^48^ Our data show that reduction in TNFα levels curbs the inflammatory response in the wound and reduces wound area and healing time (Figures 3b and 4). Treated wounds healed almost completely by Day 12, while untreated wounds were open as of Day 16. The untreated wounds in two of the five mice were so ulcerated that they opened again on Day 11, necessitating the sacrifice of those mice. Even for those mice, the treated wounds were healing faster than the PBS‐treated wounds and had not reached the same level of ulceration.

Although it was beyond the scope of this study, we anticipate that siRNA therapy would be best used in combination with a moisture retentive dressing. These dressings are known to accelerate wound healing in general when compared to a dry or uncovered wound bed.^17^ In addition to improved healing due to moisture in the wound, a dressing may also facilitate repeat applications of siRNA solution by softening the tissue and promoting solution uptake. The present study was limited to two applications of siRNA on Days 2 and 3, as scab formation prevented absorption of the treatment solution.

One of the challenges of studying inflammation in wound healing is the heterogeneity of the wound environment. The use of double‐wounded mice combats this heterogeneity by providing a built‐in control that accounts for mouse‐to‐mouse variation in inflammatory cytokine levels and the overall wound healing response. Our data indicate that TNFα expression was more similar among diabetic mice than among nondiabetic mice, differing by less than a factor of 2, rather than a factor of 3. Nanoparticle‐treated wounds in nondiabetic mice also did not heal significantly faster than control wounds, at least in the short‐term RNA interference study. This was not surprising, however, because nondiabetic mice do not suffer from an overproduction of TNFα that impedes healing. While normoglycemic mouse wounds halve in size by about 2–3 days, diabetic mice may require weeks to reach the same point.^55^

## 5 CONCLUSION

Diabetes can have several deleterious consequences, including impaired wound healing and the formation of a chronic foot ulcer. Unlike current treatments for chronic foot ulcers, which are largely palliative, RNA interference therapy addresses the underlying cause by reducing the chronic inflammation in the wound. Topical application of siTNFα‐loaded LNPs reduced TNFα expression in nondiabetic wounds by 54% at a 500 nM dose, whereas 250 nM effected 43% gene silencing in the more clinically relevant diabetic mouse model. Treated diabetic wounds closed within 13 days, which was statistically faster than control wounds, which remained open on Day 16. Together, these data highlight the potential of siTNFα‐loaded LNPs as an alternative therapeutic to address chronic inflammation, one of the major biological irregularities endemic to the diabetic wound.
